# The role of Virus "X" (*Tortoise Picornavirus*) in kidney disease and shell weakness syndrome in European tortoise species determined by experimental infection

**DOI:** 10.1371/journal.pone.0210790

**Published:** 2019-02-19

**Authors:** S. Paries, S. Funcke, O. Kershaw, K. Failing, M. Lierz

**Affiliations:** 1 Clinic for Birds, Reptiles, Amphibians and Fish, Faculty of Veterinary Medicine, Justus Liebig University, Giessen, Germany; 2 Department of Veterinary Pathology, Freie Universitaet Berlin, Germany; 3 Unit for biomathematics and Data Processing, Justus Liebig Universitiy, Giessen, Germany; Instituto Butantan, BRAZIL

## Abstract

*Tortoise Picornavirus* (ToPV) commonly known as Virus "X" was recently discovered in juvenile European tortoises suffering from soft carapace and plastron as well as kidney disease. Therefore, this virus was a potential candidate to be a causative agent for these disease patterns. Spur thighed tortoises (*Testudo graeca*) seemed to be more susceptible to establish clinical symptoms than other European species like *T*. *hermanni*. Thus this trial investigated the role of ToPV in the described syndrome. Two groups of juvenile European tortoises (*T*. *graeca* and *T*.*hermanni)* each of 10 animals, were cloacally, oronasally and intracoelomically inoculated with an infectious dose (~ 2000 TICD) of a ToPV strain isolated from a diseased *T*. *graeca*. A control group of two animals of each species received non-infected cell culture supernatant. The tortoises were examined daily and pharyngeal and cloacal swabs for detection of ToPV-RNA by RT-PCR were taken from each animal every six days for a period of 6 months. At the end of the study the remaining animals were euthanised and dissected. Bacteriological and parasitological tests were performed and organ samples of all tortoises were investigated by RT-PCR for the presence of ToPV and histopathology. Animals that were euthanised at the end of the experiment, were examined for presence of specific anti-ToPV antibodies. Several animals in both inoculated groups showed retarded growth and a light shell weakness, in comparison to the control animals. Three animals were euthanised during the trial, showing reduced weight gain, retarded growth, severe shell weakness and apathy, in parallel to clinical observations in naturally infected animals. In all inoculated animals of both species an intermittent virus shedding, starting from 18 days post inoculation (d.p.i.), till 164 d.p.i. was detected, while the control animals remained negative. The virus was successfully reisolated in terrapene heart cell culture in 16 of 20 inoculated animals of both species. Histopathology of most inoculated animals revealed a lack of bone remodeling and vacuolisation in kidney tubuli which supports the described pathogenesis of nephropathy and osteodystrophy. Anti- ToPV antibody titres ranged from 1:2 to >1:256 in 13 of 20 animals, whereas all control animals were seronegative. The study proofed the Henle Koch`s postulates of ToPV as causative agent for shell dystrophy and kidney disease in both *testudo* species. The proposed species specific sensitivity towards clinical disease was not observed.

## Introduction

In a collection of captive European and African tortoises, that included the species *Testudo graeca*, *T*. *hermanni*, *T*. *marginata*, *T*. *horsfieldii*, *Chelonoidis carbonarius*, *Centrochelis sulcata*, *Geochelone elegans*, *Stigmochelys pardalis*, *Astrochelys radiata* and *Aldabrachelys gigantea* a novel virus named "Virus X" was detected [1]. As some of the animals suffered from shell weakness, and after ruling out other common causes, the virus was proposed as a causative agent for these signs in juvenile tortoise hatchlings [1,2]. Soft carapace and plastron are frequently observed in captive chelonians. Tortoises are often seen with soft shells followed by anorexia, apathy and death. Primarily these signs are caused by the lack of UVB light (from inadequate housing) and dietary imbalances, including a low calcium intake, imbalanced calcium phosphate ratio and high levels of protein and oxalic acid intake. Parasite infestation with *Hexamita parva*, a protozoal agent that causes kidney failure in the final stage, bacterial infections and kidney failure can be other causes for shell softening [3,4,5,6]. While these causes were ruled out, some cases remained unclear in a collection that was investigated for shell softening, especially in the hatchlings of *T*. *graeca* that died approximately six weeks after the occurrence of the first symptoms. The carapace and plastron demonstrated a greyish colouration and were easy to bend with pressure administered by a finger. Anorexia and apathy occurred shortly before death [1,7]. Histopathologic evaluation revealed hepatic hemosiderosis, hypoplastic anaemia, osteodystrophic changes in the carapace and the skeleton and congestive glomerulonephrosis in the kidney [1].The bony layer of the carapace was characterised by a predominance of fibrous tissue containing thin discontinuous strands of osteoid [1]. Physiologic bridging between the bone plates was absent [1]. All common causes for soft shell disease were ruled out by clinical examination, bacteriological and mycological cultures were investigated and a parasitological investigation of faeces was performed [8,9]. Housing was appropriate with outdoor cages and a diet of fresh greens, hay and vegetables. Supplementation with a formula of minerals and vitamins (Korvimin, WDT,Germany) was adequate for these tortoises [1,6,7]. All listed investigations did not reveal specific reasons for the cause of shell softening and the rapid decrease in that population. A viral agent was suggested and conjunctival, pharyngeal and cloacal swabs were taken for virus isolation on terrapene heart cell culture (TH-1, ATCC CCL-50). The isolated virus demonstrated a cytopathic effect including cell lysis and cell rounding. It was characterised as a non-enveloped, small, single stranded RNA virus with an icosahedric capsomer, and classified as a member of the family *Picornaviridae* pipeline-assay [1,7]. It was named *Tortoise Picornavirus (*ToPV*)*. For viral detection, a RT- PCR pipeline was established [2]. However, this virus was also detected in healthy tortoises of the same collection, as well as in other studies that included healthy animals [10,11,12,13,14] Heuser et al. [1] isolated the virus from different species but only *T*. *graeca* and one *Geochelone elegans* demonstrated clinical signs, whereas other species showed normal development, but evident seroconversion. The virus had also been detected recently in other species, and the genome sequence was determined and analysed for placing the virus in the phylogenetic pattern [15]. In August 2017 it was classified as *Torchivirus* by the International comettee on Taxonomy of Viruses (ICTV) [16]. Recent investigations considering the genetic variability using varying PCR protocols with different primer pairs revealed divergent strains [13,15]. The causative role of ToPV in the described disease complex remained unclear.

This study was designed to proof the Henle Koch`s Postulates, and to compare the pathogenesis with the findings of the previous investigations in naturally infected tortoise collections and to evaluate the susceptibility of two different European tortoise species to ToPV.

## Material and methods

This study was approved by the Ethical board Regierungspräsidium Giessen, State of Hesse, Germany.

Approval number: V54-19c2015h01GI18/9 Nr.10-12

### Animals and housing

Twenty four juvenile European tortoises (*Testudo graeca (*n = 12*) and Testudo hermanni (*n = 12*)*) were obtained from four different ToPV negative collections (tested repeatedly by PCR and serology) at the age of 2 to 14 days. Each hatchling was tested negative for ToPV- RNA by RT-PCR three times over an interval of one week. X-ray examination of each animal was performed and swab samples were taken from the pharynx and cloaca. The samples were examined using aerobic culture and microscopic investigation to rule out bacteria, fungi and parasites known to cause shell softening, such as *Hexamita parva*, bacteria that cause nephritis and oxyurid nematodes that cause enteritis. The animals were housed in a 22–25°C room in cages containing a soil and sand mix as bedding and several hiding places. UV light (Osram Vitalux 300 Watt, twice daily for 30 min) and heat sources, that provided a hot spot of 40–45°C for 10 hours a day were installed in the cages. Humidity was kept at 50–70% by moistening the soil. All parameters were measured digitally twice a day. The animals were fed daily with hay, fresh greens (dandelion, rocket and local herbs) that were supplemented with Korvimin (WDT Germany) every second day. Water was available *ad libitum* and refreshed daily. The weight of each animal was measured prior the experiment.

### Inoculum

The virus for inoculation originated from the tongue of a *Testudo graeca* presenting severe shell softening at the age of six to eight weeks and died after a short period of anorexia and apathy [7]. It was characterised as ToPV by sequence analysis [7].The virus was passaged on three day old TH-1 cell culture and harvested after five days of incubation at 28°C. Cells were then scraped and the supernatant was sonified by 3 x 60 sec. pulse (Branson Sonifer B-15, Branson sonic Power, Danbury, CO, USA), clarified by centrifugation at 670 g for ten minutes (Hettich Rotanta, Tuttlingen, Germany); the supernatant was then titrated, diluted and aliquoted for inoculation, with an infectivity titre of 10^4^TCID_50_ per ml.

### Experimental infection of tortoises

**Fig 1:**
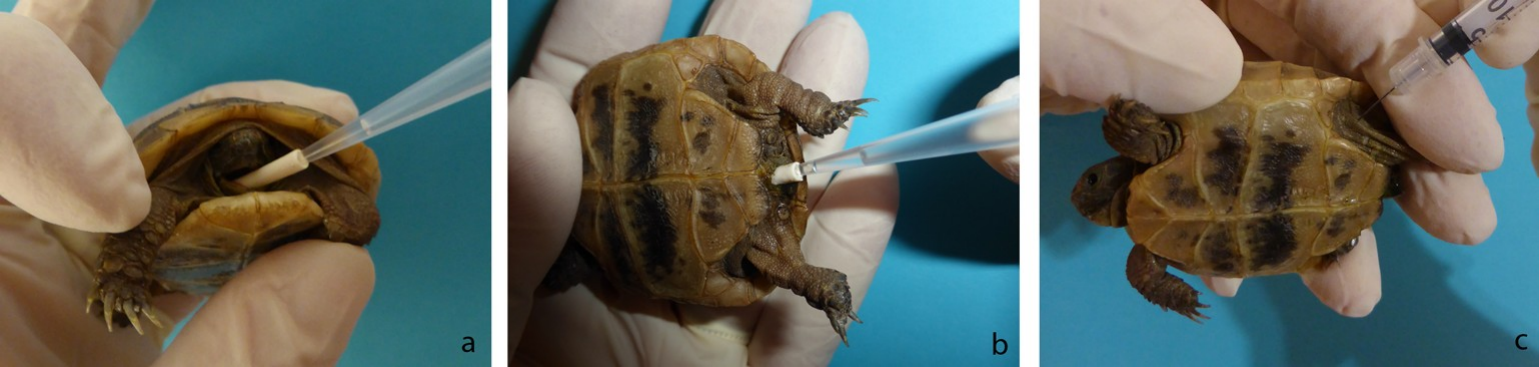
Inoculation of the tortoises. a: oronasally; b: intracloacally; c: Intracoelomically; here *T*. *graeca*.

The animals were devided into two infection groups of 10 animals from each species (group1:*T*. *graeca*; group 2:*T*. *hermanni* as well as a control group (group 3) with two animals from each species. The required animal count for each group was statistically determined using the program BiAS for windows, version 9.08, based on the approach that the infection would either be successful or that the animals would show no clinical signs (error margin α = 0,05; β = 0,1). All animals were weighed prior to and every second day post infection and housed under similar conditions. The control animals were kept in a separate room and were always handled first. Group 1 and 2 were inoculated oronasally (o), cloacally (cl) and intracoelomically (ico) with 0.066 ml of the above described inoculum per infection route, meaning a total amount of 0.198 ml (~2000 TICD_50_) inoculum per animal. All inoculations were processed without sedation or anaesthesia and performed either with a pipette (o, cl) or via injection with a syringe and 22G needle (ico) (Fig 1).The four control tortoises of group 3 received the same amount of non-infected cell culture supernatant in the same ways as the two other groups.

### Clinical course and sampling

Over a period of 164 days post inoculation (p.i.) the tortoises were examined daily for clinical signs including weight loss, anorexia, apathy and discoloration of the plastron. Weight was recorded and firmness of the shell was measured by gentle pressure with one finger every two days when the animals were handled for sampling, Methods of automatically measuring firmness in order to have a more objective evaluation were not practical due to the small size of the animals. Pharyngeal and cloacal swab samples were taken from each animal every six days prior feeding. In cases of severe clinical signs, such as no food intake, immobility and shell softening of the carapace and plastron for more than three days, the animals were euthanised. Samples were obtained by rotating a cotton tip applicator along the mucosa of pharynx, cloaca and conjunctiva. They were stored at -80°C immediately.

All surviving animals including the control group were euthanised at the end of the experiment on days 164 p.i. (*T*. *hermanni*) and 165p.i. *(T*. *graeca*). All dead or euthanised tortoises were necropsied and samples of their organs (brain, tongue, heart, liver, lungs, kidneys, spleen, intestines and bone) were stored at -80°C for molecular investigation. For histopathology samples of the same organs were fixed in 5% buffered formalin, embedded in paraffin, sectioned at 5 μm and stained with hematoxylin and eosin following standard procedures. Evaluation was performed by microscopic investigation, and changes, particularly in bone and kidney samples, were rated from 0 to 3 (no alteration (0) to severe changes (3)) Fibrous and osteoid organisation, width of bone matter and the ratio of cells like osteoblasts and osteoclasts in bone samples. Furthermore the presence of vacuoles in the tubular epithelium, the interstitial space and the tubulus lumen in kidney samples were evaluated. Samples collected from the same organs were fresh frozen for RT-PCR for the detection of ToPV RNA, and for culturing on TH 1 cell culture. To exclude bacterial and fungal infection, smears from heart, liver and lungs were streaked on blood agar, followed by aerobic cultivation for 48 h [8]. Smears of the jejunum and colon were investigated for parasites by microscopic evaluation [9]. As blood sampling was not performed during the experiment, due to the low weight of the animals, blood was collected only during necropsy. Sera were separated by centrifugation and investigated for specific anti- ToPV antibodies by a virus neutralisation test.

### RT-PCR

The swab and organ samples were tested for the existence of ToPV RNA by RT- PCR according to the protocol of Keil et al. (personal communication). Frozen samples were thawed at room temperature. RNA was isolated using the Qiagen RNeasy Mini Kit (Qiagen, Hilden, Germany), and cDNA synthesis was performed using the Transcriptor High fidelity cDNA synthesis kit (Roche diagnostics, Mannheim, Germany). PCR was performed using the KOD Hotstart Polymerase Kit (Merck chemicals, Darmstadt, Germany), with the primers Kei 4343 (CTTCAGTATAGAAAACGGATTTGATG) and anchored Oligo (dT)18 (Keil et al., personal communication). Sensitivity and specificity were tested prior to investigations. PCR products approximately 800 bp in length were visualised by agarose gel electrophoresis (Agarose NEEO ultra, Carl Roth, Karlruhe, Germany), using 120 V for 35 minutes (Electrophoresis Power supply, Pharmacia, Amersham, California, USA). Isolate (ZU 1243/37) was used not only for inoculation of the animals in the experiment, but as a positive control in the other assays.[1].

### Serological examination/virus neutralisation test

Sera collected from the animals were tested for specific anti ToPV antibodies developed against the isolate ZU 1243/37 which was used in our infection study [1].

25 μl of each serum and 25 μl basal medium Eagle (BME, containing 1% streptomycin) were placed in the first row cavity of a 96 well plate (Nunc GmbH, Zürich, Switzerland) and diluted log _2_ in eight steps. Positive (isolate Ni 402) [7] and negative sera used in previous studies (Qu 06) [7] were included in each plate as well as a cell control. Each cavity-well was covered by 25 μl of the titrated virus suspension 1243/37 ZU (1000 TICD _50_) followed by incubation at 28°C for 120 min. Afterwards the content of this plate was transferred into a 96 well plate with confluent terrapene heart cell mono layers (TH-1, ATCC CCL-50). After the plates were incubated for another 120 min, the supernatant was discarded, and the wells were covered with 100 μl of minimal essential medium (MEM HEPES containing 10% Fetal calf serum, 1% Penicilin/Streptomycin, 1% Glutamin) and incubated at 28°C. The plates were daily evaluated for confluency of the cell layer and cytopathic effects (CPE) like cell rounding and cell lysis. Evaluation was ceased 6 days post inoculation if no CPE was seen. The titre was expressed as the inverse of the highest dilution in which a CPE was seen. A titre >1:2 was regarded as positive.

### Virus isolation

For virus isolation, swab- and tissue samples were incubated in MEM HEPES with 1% Penicillin/Streptomycin (Biochrom AG, Berlin) for four hours at 4°C, sonified three times with 60 sec. of pulsing and centrifuged at 670 g. 0.2 ml supernatant was used for inoculation in 2 ml petri dishes of two- day- old confluent TH1 cell monolayers. Filtering of homogenised tissue samples with a 0.45 μm filter (Biofiltronic, Nörten Hardenberg, Germany) was needed to avoid bacterial and fungal contamination of the cultures. Cell cultures were incubated at 28°C and observed for CPE for 6 days. Cultures showing a CPE were harvested, cultures with no CPE after 6 days were passaged in the same way. If no CPE was observable after three passages, the samples were regarded negative.

### Statistics

The statistical analysis was performed with the statistical program packages BMDP [17] as well as StatXact and LogXact [18]. For each variable analysed, the significance level was set to α = 0.05.

The statistical analysis of body mass was performed to evaluate the course of mean mass change in the inoculated animals compared to the control animals. This was calculated by using a three-way analysis of variance (ANOVA), because body mass was nearly normally distributed on the single measure points. The ANOVA includes the factors “infection” and “tortoise species”, as well as repeated measures in terms of time, using the program BMDP2V. The divergent course between infected and non- infected animals is hereby statistically evaluated by the interaction of the effect of inoculation of the tortoises and the time effect. For describing the data, the arithmetic mean as well as the standard deviation of the body mass on the 26 repeated measures were calculated every six days post inoculation.

The other target variables in this study were the duration of virus detection in the swab samples during the trial, as well as the duration of shell softening in the carapace and plastron. Based on the indication of early euthanasia in those animals showing severe clinical signs and therefore different time of observation for single animals, these absolute values were transformed into relative values (relative virus detection period (relVDP) and relative shell softening time for the carapace (rel SSTC) and plastron (rel SSTP)). The correlation between those variables was statistically evaluated using a correlation analysis (BMDP6D) and the strength of the non-linear but monotone relation was evaluated by using spearman´s rank correlation coefficient (Program BMDP3S).

As a further endpoint the degree of severity in the histological alterations of kidney and bone were recorded and were evaluated in grades from 0 to 3. The correlation between relative time values and the degree of severity was analysed by spearman´s rank correlation coefficient. Due to several ties in the data the exact method was used (program StatXact). In regards to the biological background, a one-sided statistical test was assumed, which means only positive correlations were considered relevant.

To evaluate the association between histological alteration in the kidneys and bone and the relative soft shell duration carapace (relSSTC) and relative soft shell duration plastron (rel SSTP), calculations were also based on exact spearman´s rank correlation coefficients in a one-sided test. This was also performed with the program StatXact.

As a consequence of severe clinical symptoms an early euthanasia was necessary in a few cases. For the analysis of this frequency in dependency on the relative VDP and the relative SSTC/SSTP, an exact logistic regression was calculated using the program StatXact.

## Results

### Clinical findings

**Fig 2:**
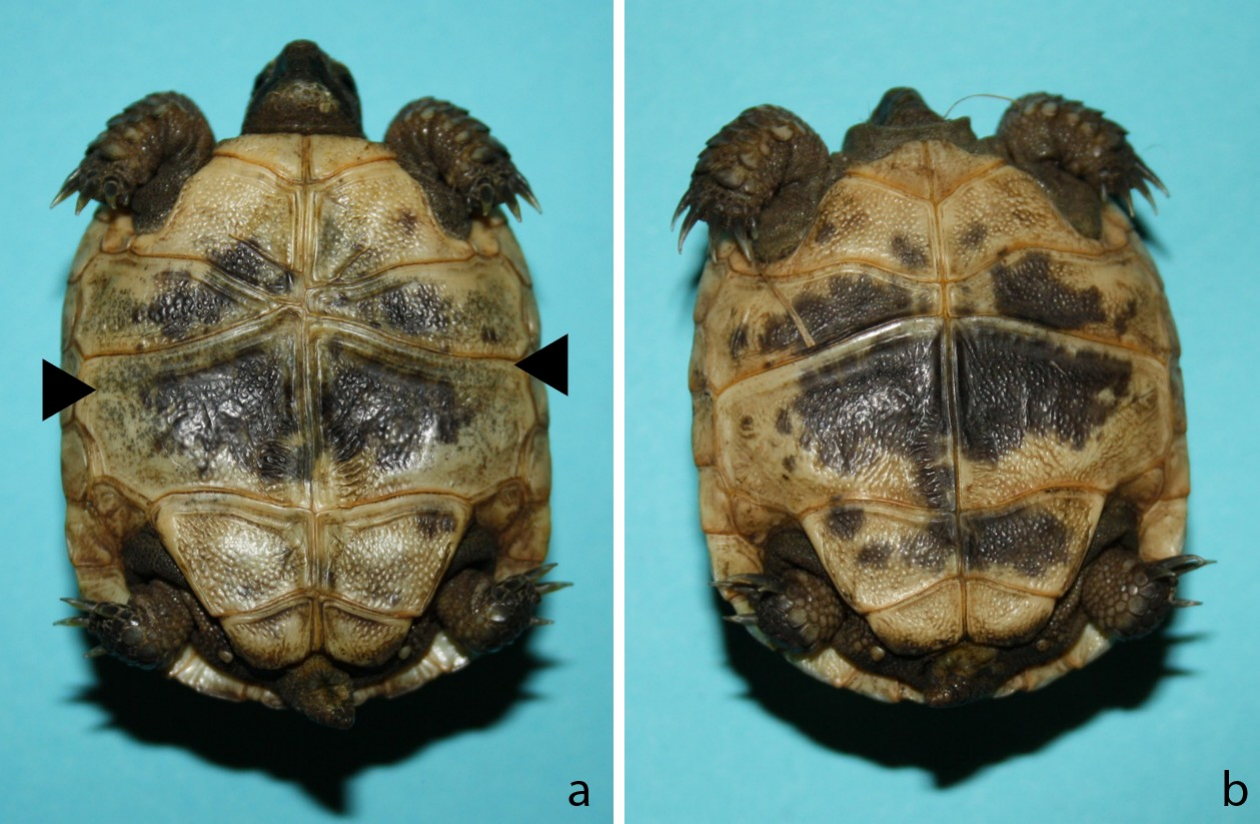
Ventral view at *T*. *graeca* no. 8 (a) and no. 9 (b) at day 90 p.i.; note the greyish colouration of the lateral plastron in G8.

**Fig 3:**
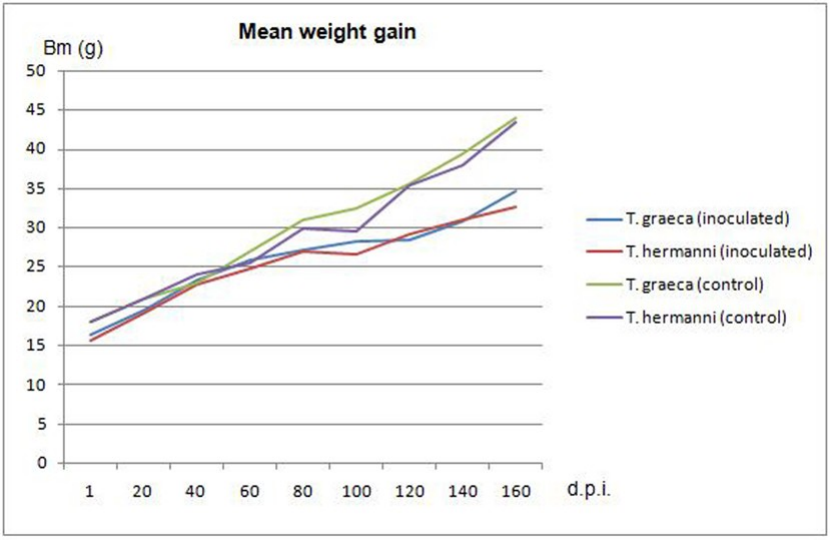
Mean weight gain in the groups during the survey period.

During the trial the infected animals were compared to the control animals of their respective species. Animals of Group 1 (*T*. *graeca*) developed healthily and actively until day 90 p.i.; two animals G8 and G9 then displayed a light softness of the plastron, extending to the carapace within 30 days. Additionally, animal G8 presented a greyish discolouration of the plastron (Fig 2). Until day 120 p.i., food intake and activity remained steady, before weight stopped increasing and activity was reduced. Both animals were euthanised at day 155 p.i. and presented anorexia and no weight gain, lethargy and a softness of the whole shell. Seven animals (G1, G2, G5, G7, G10 including the two symptomatic animals G8 and G9) gained only 81.7% weight (mean weight gain in terms of the original weight), during the survey period. In comparison the remaining three *T*.*graeca* displayed a mean weight gain of 127.5% and the two control animals showed a mean weight gain of 149% in the same time interval (Fig 3). The animals G1, G5, G7 and G10 presented the beginnings of shell softening around day 100 p.i., but displayed firming of the shell towards the end of the survey period (165 days), other clinical symptoms were not detected.

Group 2 (*T*. *hermanni*) developed normally until day 67 p.i., on this day, animal H3 demonstrated closed eyes, reduced activity and no food intake. Shell softening was first detectable on the ventral shell and extended dorsally and H3 was euthanised on day 108 p.i. The animals H4, H7, H8, H9, H10 in addition to H3 showed retarded growth and slow weight gain (mean weight gain of 53.9%) compared to H1, H2, H5 and H6 (mean weight gain of 117.9%) as well as the control animals (mean weight gain of 148.9%) (Fig 3). Some animals (H1, H4, H8, and H9) that presented slightly soft shells did not exhibit clinical symptoms and their shell began to get firm towards the end of the trial as seen in the infected *T*. *graeca* with mild shell softness as well.

### Macroscopic lesions and histopathological findings

**Fig 4:**
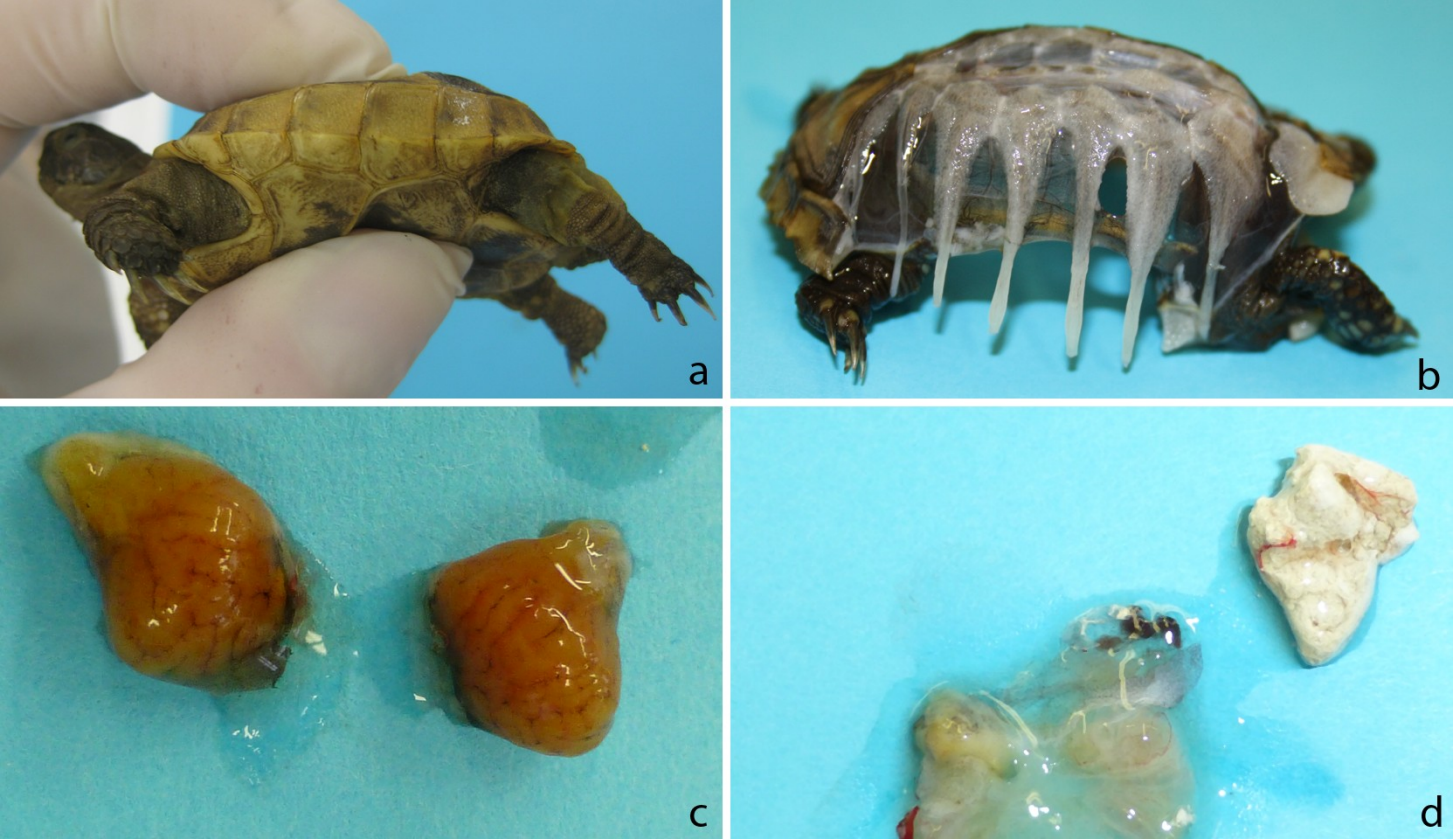
Gross pathological findings in the euthanised animals. a: soft carapace and plastron b: plastron with sieve like bone matter and lack of bridging between the ribs c: enlarged swollen kidneys d: urinary bladder emptied, with uric acid conglomerates.

**Fig 5:**
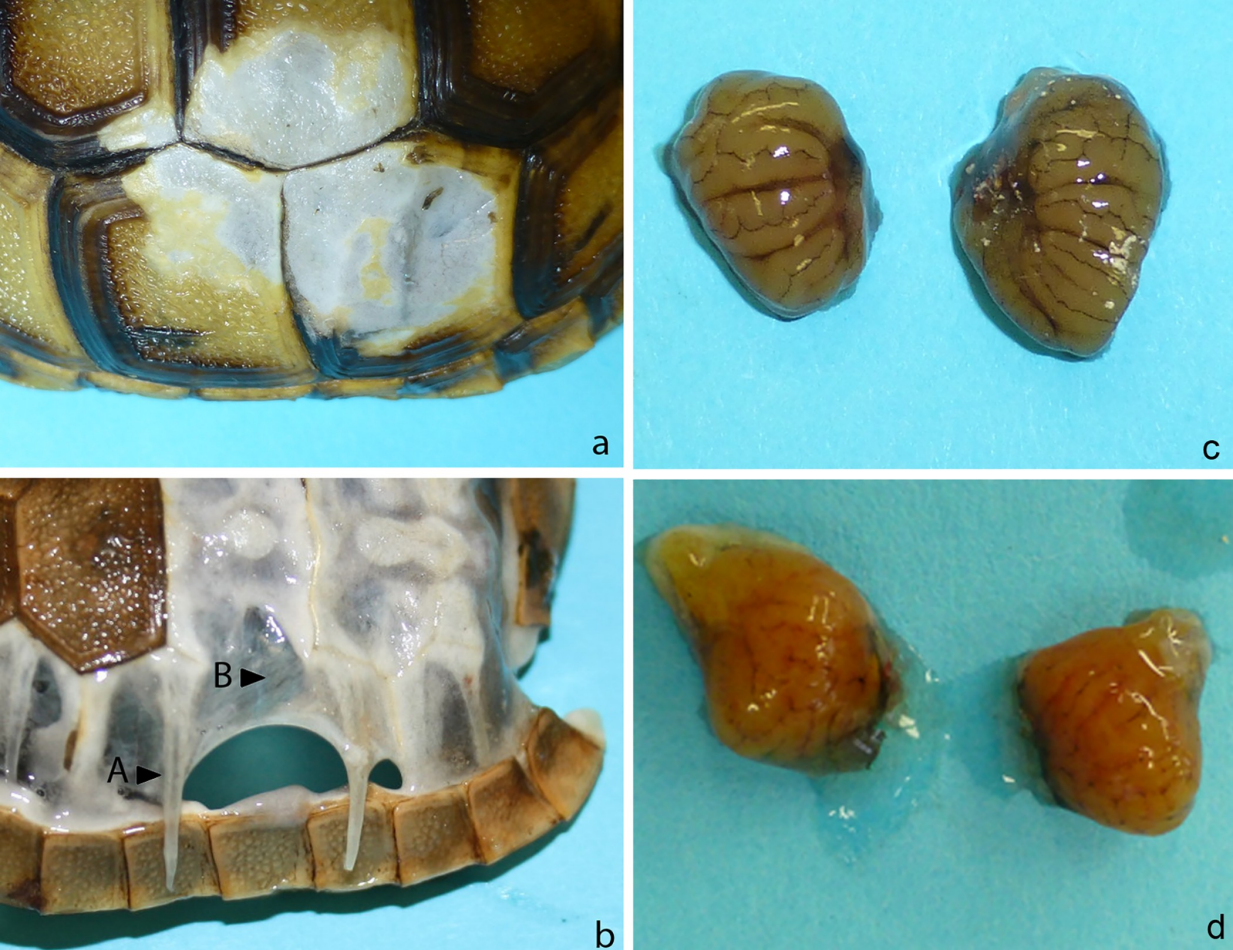
Comparison of carapace structure and kidneys in infected and non-infected tortoises. a: Carapace of control animal *T*. *graeca CG1*; b: Carapace of animal *T*. *graeca* G9; c: Kidneys of control animal *T*. *graeca* CG1 d: Kidneys of *T*.*graeca* G9 The carapace of G9 shows only thin rib bones (A) with fibrous tissue in between (B), in the control animal the intercostal space is filled with bone matter The kidneys of the infected animal are swollen and the natural structure is not visibleas in the kidneys of the control animal.

In the three tortoises euthanised during the trial (G8, G9, H3), necropsy detected thin soft rib bones with poor fibrous tissue in the intercostal regions and single bone islets with a perforated structure (Figs 4B and 5B). It was possible to loosen the skin layer from the bony matter in these animals. In G8 and G9, enlarged urinary bladders were presented which contained uric acid conglomerates (Fig 4D). The kidneys of these animals were enlarged and showed dark capillary markings on their capsules in comparison to the control animals (Figs 4C, 5C and 5D). In H3, the urinary bladder was also filled but the markings on the kidney capsules were absent. In both groups, animals that presented mild symptoms (3 of 10 in group 1; 4 of 10 in group 2) also displayed slightly enlarged kidneys in comparison to the control animals and the carapace and plastron bones were not forming a complete bony shell as it was seen in the control animals in both species (Fig 5A).

**Fig 6:**
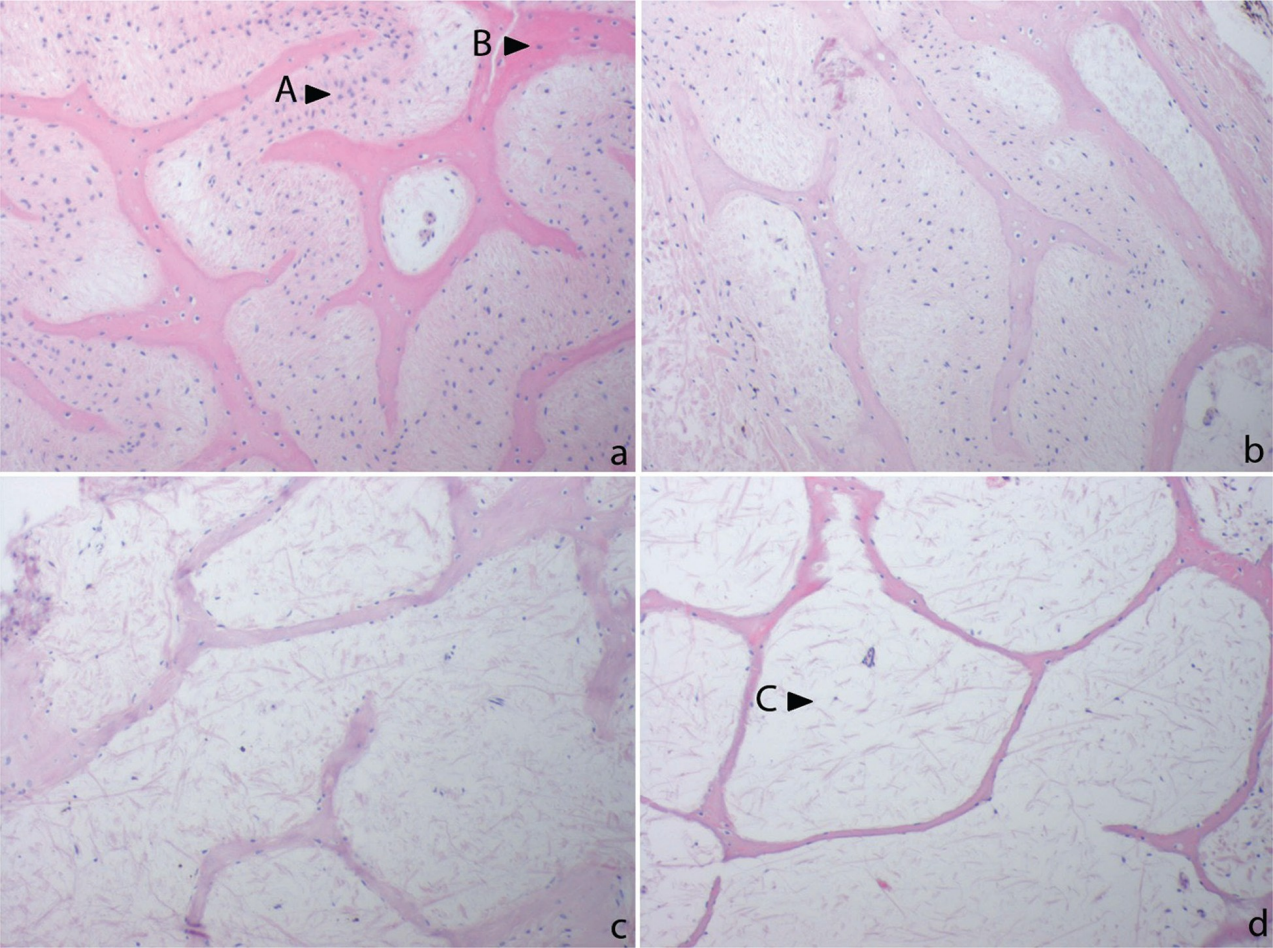
Histologic findings in the carapace bone; 200 x; HE staining. a: control animal: stage 0; densely aggregated fibrous tissue (A) with numerous fibrocytes and dense bone matter with numerous osteocytes (B) b: stage 1; fibrous tissue forming irregular aggregates, variable density of fibroblasts, bone matter with reduced number of osteocytes c: stage 2;moderate rarefication of fibrous tissue with fiber separation and substantially reduced numbers of fibrocytes; bone matter with loosened structure and reduced numbers of osteocytes d: stage 3; severe rarefication of fibrous tissue with only occasional fibrocytes (C), extremely narrowed bone spicules with only scattered osteocytes.

**Fig 7:**
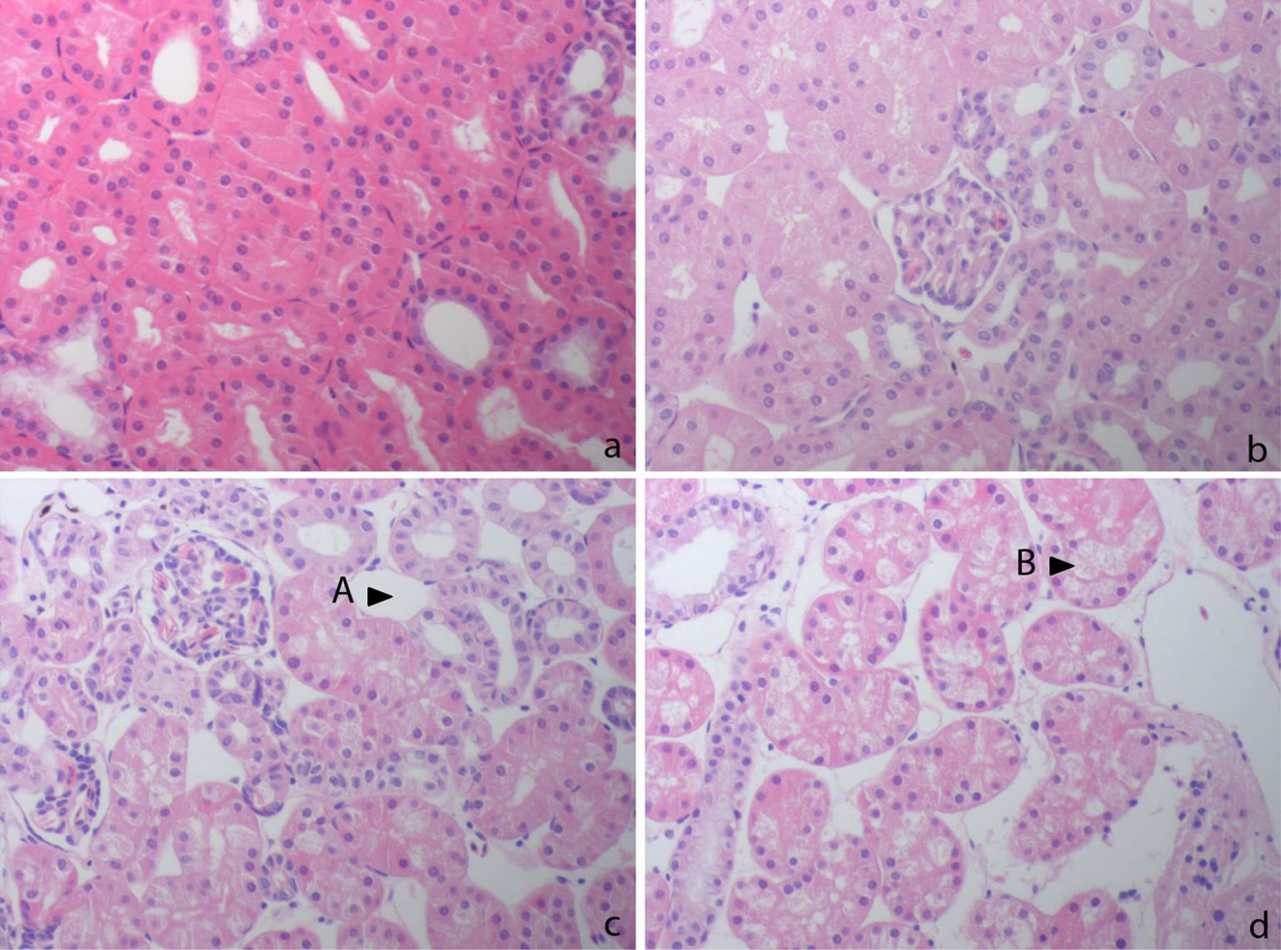
Histologic findings in the kidneys; 200 x; HE staining. a: Control animal, stage 0; no alterations in kidney parenchyma b: stage 1; mild interstitial edema, mild tubular vacuolization c: stage 2; moderate tubular vacuolisation, moderate interstitial edema (A) d: stage 3; severe tubular vacuolisation (B), tubular lumina are absent, severe interstitial edema.

Histopathologic examination of the animals G8, G9 and H3 revealed alterations in the bone and kidney tissues (Figs 6 and 7). Samples of the carapace and plastron were dominated by loosely arranged cells andpoor fibrous tissue with only few narrowed bone trabeculae containing only scattered osteocytes. Signs of bone remodelling were absent (Fig 6B–6D). Animals that solely presented slow weight gain and only mild clinical symptoms (*T*.*graeca*, no. *1*, *2*, *5*, *7*, *10* and *T*. *hermanni*, no. 4, 7, 8, 9, 10) showed a higher density of the fibrous tissue with more fibrocytes and an overall increased thickness and bridging of bone trabeculae, but only low osteocyte density with few osteoclasts being detectable. Animals that presented normal weight gain (*T*. *graeca*, no. 3, 4, 6; *T*. *hermanni*, no. 1, 2, 5, 6) and control animals both displayed continuous, osteocyte rich and branched bone trabeculae separated by dense fibrous tissue with numerous fibrocytes (Fig 6A).

The kidneys of the diseased G8, G9 and H3 presented interstitial edema and vacuolisation of the tubuli; the tubular lumina were narrowed in the strongly vacuolised areas (Fig 7C and 7D). In comparison, in the kidneys of clinically healthy animals that showed retarded growth (*T*.*graeca*, no. 1, 2, 5, 7, 10 and *T*. *hermanni*, no. 4, 7, 8, 9, 10) the tubular epithelium was unchanged with only little interstitial space and occasional cytoplasmic vacuoles (Fig 7B). The control animals as well as animals with normal weight gain (*T*. *graeca*, no. 3, 4, 6 and *T*. *hermanni*, no. 1, 2, 5, 6) presented homogenous kidney parenchyma (Fig 7A)

For comparison, the alterations were grouped into different degrees (0/1/2/3—no visible alteration/ mild/ moderate /severe; Table 1).

**Table 1:** Post mortem findings in experimentally *tortoise picornavirus* infected tortoises.

ID	Antibody titre p.m.*	RT- PCR positive organs	Histopathology**		Infectious virus***
	
	(log _2_)		Bone	Kidney	
		Group 1			
G1	32	T, Lu, Kd, Sp, I	1	0	+
G2	4	Br, T, Lu, Kd, Sp, I	1	0	+
G3	>256	Br, T, Lu, Sp, I, Bo	0	0	+
G4	64	T, Sp	0	1	+
G5	< 2	Br, T, Bo	2	2	+
G6	2	Sp, Bo	2	2	+
G7	< 2	Sp, Bo	2	2	+
G8-	< 2	T, Kd, Bo	3	3	+
G9-	< 2	T, Kd, Sp, Bo	3	3	+
G10	4	T, Kd, Sp, Bo	2	3	+
		Group 2			
H1	8	T, H, Kd, Bo	0	1	+
H2	2	T, Kd, Bo	0	1	+
H3-	< 2	T, Bo	3	3	+
H4	< 2	T, Kd, I, Bo	1	1	+
H5	32	T, Kd, Sp, I	0	0	+
H6	>256	T, Li, Kd, Sp, I, Bo	0	0	+
H7	32	T, Kd, Sp	0	2	+
H8	8	T, Li, Lu, Kd, Sp, I, Bo	2	3	+
H9	< 2	Sp, Bo	2	3	+
H10	64	H, Li, Sp, I	1	2	+
		Group 3			
HK1	< 2	no	0	0	no
HK2	< 2	no	0	0	no
GK1	< 2	no	0	0	no
GK2	< 2	no	0	0	no

G = *Testudo graeca*, H = *Testudo hermanni*, K = control animal either G or H

*antibody titers < 1:2 = negative, p.m. = Post mortem

Organs: Br = brains, T = tongue, H = heart, Li = liver, Lu = lungs, Kd = kidneys, Sp = spleen, I = intestine, Bo = bone

**0 = None, 1 = Mild, 2 = Dominant, 3 = Severe

***Re-isolation of infectious *Tortoise picornavirus* in TH 1cell culture

- animals with early euthanasia

Bacteriological, mycological and parasitological investigation of organ swab samples:

None of the animals showed aerobic bacterial and mycological growth in heart-, liver- or lung-swabs.

In the smears of the intestinal parts of animal H3, two oxyurid eggs were found but no adult worm stages were detected. None of the remaining animals showed any parasitic infestation.

### Tortoise Picornavirus—RNA detection during the trial

**Fig 8:**
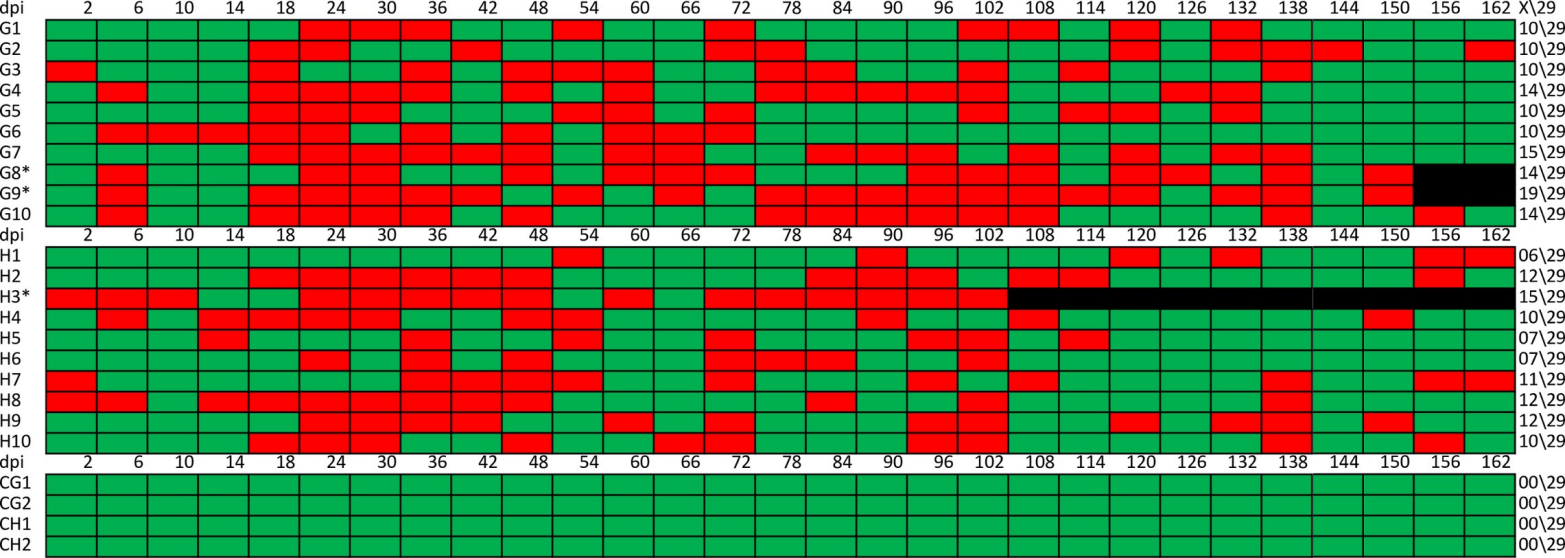
Number of positive RT PCR tests of swab samples during the trial; total test amount 29 G: *T*. *graeca*; H: *T*. *hermanni*; 1–10: Number of single animal; dpi: Days post inoculation Red: Positive; Green: Negative; Black: Not tested Last column presents positive results of total testing (29) in each animal, to show the high number of positive tests in early euthanized animals.

ToPV- RNA was detected in the swab samples of all inoculated tortoises, while the control animals remained negative. Shedding of Virus RNA was intermittent and followed no specific pattern. Two days post inoculation, the animals started shedding ToPV-RNA, which might have been the inoculated virus. This ceased in most animals until day 18 p.i., when shedding started again. (for detailed results please see Table 1 and Fig 8). In the animals that were euthanised during the trial, virus shedding was detected more frequently after clinical signs were seen. For detailed results on duration of shedding see Fig 8.

### Tortoise Picornavirus—RNA detection in tissue samples

**Fig 9:**
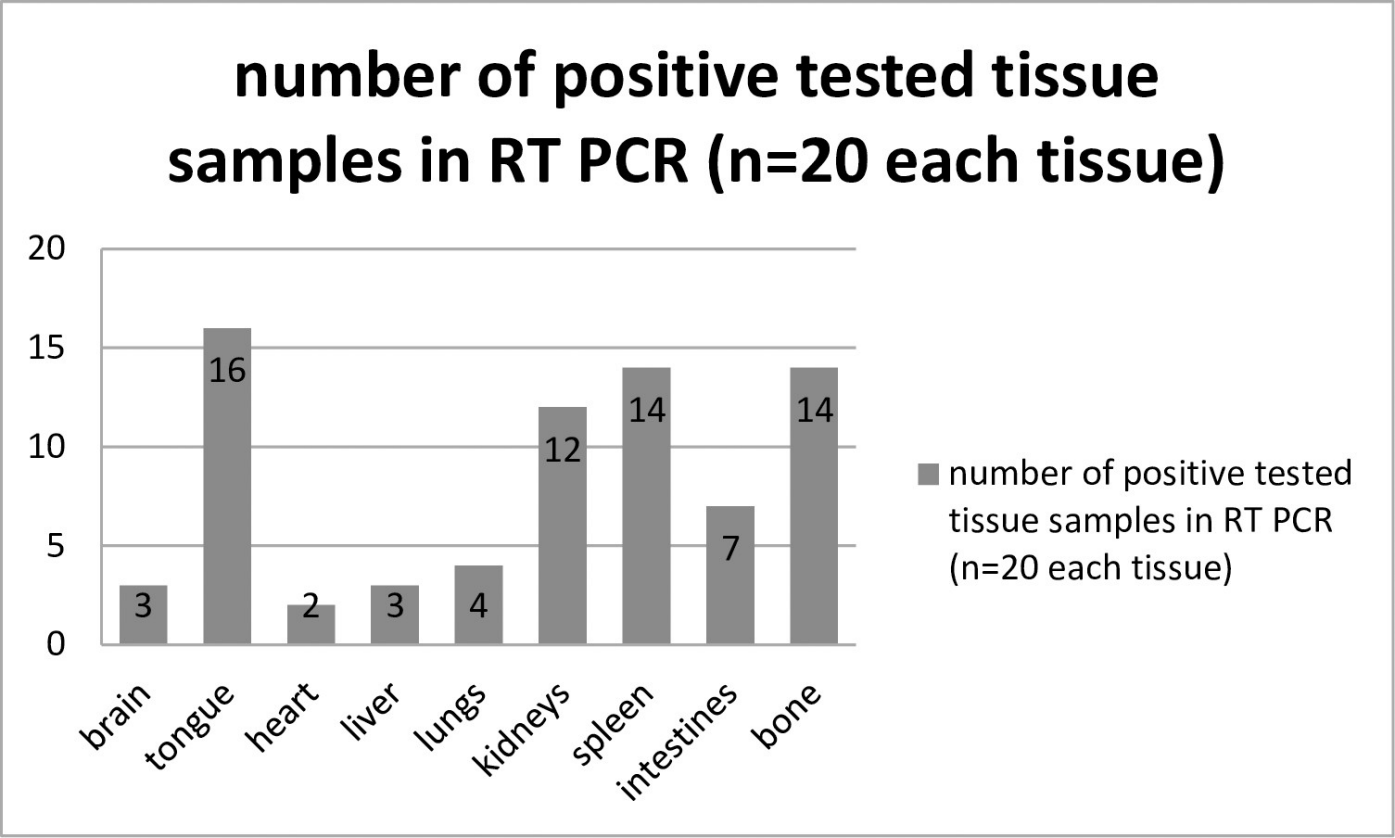
Number of RT PCR positive organ samples collected during necropsy; total number 20.

RNA of ToPV was detected in tissues of all inoculated animals (n = 20), mostly in the tongue (n = 16/20), kidneys (n = 14/20), bone (14/20) and spleen (n = 12/20). The detailed results are reported in Table 1 and Fig 9. The control animals were ToPV negative in all tissue samples.

### Virus isolation

Virus isolation was successful in organ samples from 16 of 20 inoculated tortoises of both species, but not in the control animals. In four animals isolation was not successful after three passages although ToPV-RNA was detected in tissue samples.

### Serological investigation of necropsy blood

Specific anti ToPV antibodies were detected in 6 of 10*T*. *graeca* and in 7 of 10 *T*. *hermanni*. Titres ranged from 1:2 to > 1:256. The two animals G3 and H6 that showed the highest titres of >1:256, were clinically healthy and had a high weight and firm carapace, whereas the animals that had no antibody titres were the ones that were euthanised earlier or showed retarded growth and severe soft carapace and plastron (G8, G9, H3, G5, and H9). The control group remained negative for the presence of specific antibodies (Table 1).

### Statistics

Statistical analysis showed a mean body mass increase in the infected animals of 15.6 g ± 8.4 g in *T*. *hermanni* and 17.3 g ± 5.5 g for *T*. *graeca*. In contrast, the mean body mass increase in the non- infected animals measured 25.5 ± 9.2 g in *T*. *hermanni* and 25.5 ± 5.0 g in *T*. *graeca*. This indicates a significant lower body mass increase for the infected animals and therefore a definite afflicted weight gain and growth compared to the non- infected animals (p = 0.0001). A statistically significant influence of tortoise species on body mass course was not observed, neither as a main effect nor as an interaction with the other potential influencing factors.

The correlation between the “relative shell softening time for the plastron” (rel SSTP) and the relative virus detection period (rel VDP) revealed a rank correlation coefficient of 0.177 combined with a p-value of 0.288- therefore a no statistically significant correlation between these variables. In contrast the “relative shell softening time in carapace” (rel SSTC) in relation to relVDP demonstrated a significant positive correlation with a rank correlation coefficient of 0.590 and a p-value of 0.0015. Therefore a correlation between shell softening of the carapace and the frequency and duration of virus detection could be proved.

With a rank correlation coefficient of 0.595 and a p-value of 0.0034, this calculation implies that the relation between the degree of histological alterations in the kidney and the relative VDP is significantly positive correlated. Likewise the correlation between the histological alterations in the bone tissue and the relative VDP showed a positive rank correlation coefficient of 0.686 combined with a p-value of 0.0006. This shows that the more frequently the virus was detected in swab samples, the severity of histological alterations in bone and kidney tissue increased. Similarly the values for the relative SSTC and the relative VDP support these results. For the correlation between the alteration in kidney tissue and the relative SSTP a rank correlation coefficient of 0.528 with an exact p-value of 0.0092, and for the relative SSTC a rank correlation coefficient of 0.643 and an exact p-value of 0.0009 were calculated, which implies a significant correlation between those variables. In terms of changes in bone tissue, a significant correlation between the relative PEDP (correlation coefficient = 0.473; p-value = 0.0177) and relative SSTC (correlation coefficient 0.525; p-value 0.0175) was detected as well. The calculations support the thesis that the longer the shell softening in carapace and plastron were present, the more severe alterations in kidney and bone tissue were detected, therefore proving a correlation between shell softening and pathological tissue change.

The analysis of early euthanasia resulting from severe clinical signs dependent on the relative time intervals of rel VDP/relSSTC/relSSTP produced significant positive relationships between the rel VDP (p-value 0.0035) and rel SSTC (p-value 0.0018). The longer those time intervals were measured, the higher the propability that the animals developed clinical signs and were euthanised. After the evaluation of the analysed data a dependency for relSSTP was not proven.

## Discussion

In our study we were able to reproduce the clinical signs of soft carapace and plastron in three animals, as previously reported for animals infected with *Tortoise Picornavirus* in previous cases [1]. Additionally, we detected animals in both inoculated groups that displayed retarded growth and mild shell softness, as well as animals that did not display any clinical signs in comparison to the control animals, but mild histologic alterations. Statistical analysis showed a significantly lower mean body mass increase in the infected animals in contrast to the mean body mass increase in non- infected animals and therefore we observed definite afflicted weight gain and growth compared to the non- infected animals (p = 0.0001).

Our histological findings parallel the results of a previous study [1], describing a field infection and therefore fullfill the Henle Koch`s postulates, as the virus could have also been re-isolated. We propose three clinical presentations: those with severe shell softness and progressive disease, animals with retarded growth and mild soft shells, and animals with no clinical presentation. Factors influencing the progression of this disease complex are yet unidentified, although the age of the animals inoculated or individual factors may influence the susceptibility.

In comparison, the animals in the previous study from Heuser displayed first symptoms at the age of 6–8 weeks after hatching and the majority died 10 to 14 days later; single animals displayed retarded growth and improved later on [7] as seen in our study as well. Since the animals in our study were inoculated after hatching they had a slightly higher age than in Heusers observations (6 weeks older). This might explain the later onset of clinical signs here, or the low number of animals with severe disease. Furthermore this might explain the lack of clinically diseased adults for this disease complex, as it was described before [1], which may instead serve as carriers for ToPV.

RT- PCR detected ToPV RNA in all animals except the control group. Virus shedding was intermittent and no specific pattern could be noticed, except that *T*. *graeca* G8 and G9 and some of the animals that showed retarded growth, were tested positive by PCR more often than animals growing normally (Fig 8). The correlation between shell softening of the carapace and the frequency and duration of virus detection proved statistically significant. All animals showed RT- PCR positive organ samples after dissection. In the study from Heuser, virus isolation was possible from the same organ samples. Here the highest and most infective titres were seen in the brain, tongue and kidneys. Bone was not tested in this study [7]. Our results proof the suspected tropism to specific tissues, such as tongue and kidneys. Virus isolation was possible in organ samples from 16/20 inoculated animals; the samples of tongue and kidneys again had the best positive results. CPE was characterised by both cell lysis and cell rounding and it occured on day 2 p.i. Heuser reported the first CPE for day 3 p.i. in their study; in our study the lytic behaviour in cell culture is similar to different previously described isolations [1,7,10,11].

The serological investigation on the necropsy sera detected specific anti -ToPV antibodies in most animals in both species. We reported the highest titres in the animals G3 (> 1:256), G4 (1:64) and H6 (> 1:256). Those three tortoises were animals that did not present any clinical signs and showed normal weight gain and a firm carapace and plastron, with only mild histological alterations. H10 had a titre of 1:64 log_2_ and presented retarded growth, but did improve up until the end of the experiment; histological marks were only mild as well. All animals with clinical signs had not developed detectable antibodies (Table 1). It is known that antibody production in tortoises is slower than in other vertebrates [19]. Studies on ranavirus infections in tortoises showed an increase of antibodiy titres over a period of five month [20]. Futhermore tortoises showed a significantly lower antibody persistence of about eight months in comparison to other vertebrates [21]. Other studies showed a persistence of antibodies for 590 days [22]. It is also known that the immune response is not fully funtional when the tortoises hatch [23]. In different studies on tortoise and snakes antibody production and seroconversion was reported to be establihed in four days to 15 weeks post- inoculation with the antigen [19,24,25,26]. It may be possible that the animals were too young to have a full immunological response to the virus, and the immune system was immature at this point [27]. The negative titre in H3 might be inherited from the short time between inoculation and euthanasia, as seroconversion might not have yet started. Other immune responses were not investigated due to the small size of the animals. Since the animals were too small for regular blood collection during the trial, the serological investigation could not be carried out regularly, so we could not proove the exsistance of any titre changes in the single animals during the survey period.

The necropsy displayed soft carapace and plastron in three animals (G8, G9, H3); these animals additionally presented slightly enlarged kidneys and filled urinary bladders containing high amounts of uric acids compared to the control animals. Either calcification of the bones did not take place in these animals or demineralisation and substitution of bone tissue through fibrous tissue set in. The histological results support our findings. Alterations in bone and kidney tissue suppose a lack of bone remodelling and tubulonephrosis. We propose tubulonephrosis and the lack of calcium uptake in the kidneys as primary causes for the severe shell softness observed. Calcium is eliminated through the urine, which is supported through the high uric acid content in the urinary bladder of the diseased tortoises. This leads to a low blood calcium level, as well as a lack of calcium for ossification processes. Due to the small size of the animals it was not possible to measure blood calcium levels. Furthermore x-rays of the animals did not reveal any useful data. Since we performed the study in juveniles, the absense of bone remodeling seems to be the major problem in these animals. Rarefication could take place in older infected animals with a firm carapace, but infection was not yet proven in these animals. Furthermore the damage of kidney tissue may cause chronical kidney disease, which may result in a later onset of clinical symptoms, or manifest in adult animals as well.

The role of the clinically healthy animals and those animals that displayed only mild shell softness with an improvement at the end of the experiment remains unclear. As histologic alterations were detectable and virus isolation was possible as well, these tortoises may function as virus carriers when introduced to new collections. In previous studies [28] on the distribution of ToPV throughout German tortoise breeders, 32% of the tested *Testudo graeca* were ToPV positive in either RT-PCR or serology, but only the offspring of one positive breeding pair presented the clinical disease. The adults remained clinically healthy. Other European tortoise species also presented positive animals, but no clinical signs were seen in juveniles. This strongly supports the limitation of clinical disease in juvenile animals, as previously described [1], and an early infection results in severe disease. The route of infection still remains unclear.

Heuser [1] suspected species specificity for *T*. *graeca*. This was not detected in our study, as both groups presented clinical symptoms as well as gross and histologic alterations of bones and kidneys. Additionally, serological results and virus isolation do not support the assumed species specificity, as *T*. *hermanni* as well as *T*. *graeca* displayed specific anti- ToPV antibodies and infectious virus in reisolation.

Species specific clinical signs as previously assumed [1] appears to not be very strict, but future investigations on other than *Testudines* should be performed.
